# Bridging cardiovascular physics, physiology, and clinical practice: Karel H. Wesseling, pioneer of continuous noninvasive hemodynamic monitoring

**DOI:** 10.1152/ajpheart.00839.2014

**Published:** 2015-02-01

**Authors:** Berend E. Westerhof, Jos J. Settels, Willem-Jan W. Bos, Nico Westerhof, John M. Karemaker, Wouter Wieling, Gert A. van Montfrans, Johannes J. van Lieshout

**Affiliations:** ^1^Edwards Lifesciences BMEYE, Amsterdam, The Netherlands;; ^2^Laboratory for Clinical Cardiovascular Physiology, Heart Failure Research Center, Academic Medical Center, Amsterdam, The Netherlands;; ^3^Department of Internal Medicine, Sint Antonius Ziekenhuis, Nieuwegein, The Netherlands;; ^4^Laboratory for Physiology, Institute for Cardiovascular Research, Vrije Universiteit, Amsterdam, The Netherlands;; ^5^Department of Systems Physiology, Academic Medical Center, University of Amsterdam, Amsterdam, The Netherlands;; ^6^Department of Internal Medicine, Academic Medical Center, University of Amsterdam, Amsterdam, The Netherlands;; ^7^Department of Vascular Medicine, Academic Medical Center, University of Amsterdam, Amsterdam, The Netherlands; and; ^8^Medical Research Council/Arthritis Research United Kingdom, Centre for Musculoskeletal Ageing Research, School of Life Sciences, The Medical School, Queen's Medical Centre, University of Nottingham, Nottingham, United Kingdom

karel hendrik wesseling (Fig. 1), emeritus professor of biomedical instrumentation, was born in The Hague, The Netherlands, on April 23, 1935. He studied electrical engineering, in his words, from 1950 on, and since 1953 at the Delft University of Technology (DUT) from which he graduated with honors in 1960. Subsequently, he worked at the DUT, did military service at the Dutch Air Force, and returned to the DUT to work in active networks. After that, he worked in radio astronomy, first in a European project and later in the United States at the National Radio Astronomy Observatory in Green Bank, WV; Charlottesville, VA; and Tucson, AZ; eventually as head of the mm-wave laboratory. In 1969, he moved back to The Netherlands “to marry a Dutch girl,” as he put it. Indeed, he married Hanny Gommers in 1971. He started working in the TNO Institute of Medical Physics in Utrecht with Professors Dick H. Bekkering and Jan E. W. Beneken and became head of the Cardiovascular Physics group in 1976. Here he developed several computational models of physiological systems, laying the basis for his later work on baroreflex, calculation of cardiac stroke volume from arterial pressure and of pressure transfer functions. His special interest was to obtain physiological parameters in a noninvasive manner. In 1984, he brought his group, now called TNO Biomedical Instrumentation, to the Academic Medical Center of the University of Amsterdam. In 1990, he was appointed professor in the Faculty of Electrical Engineering of the Eindhoven University of Technology.

**Fig. 1.**
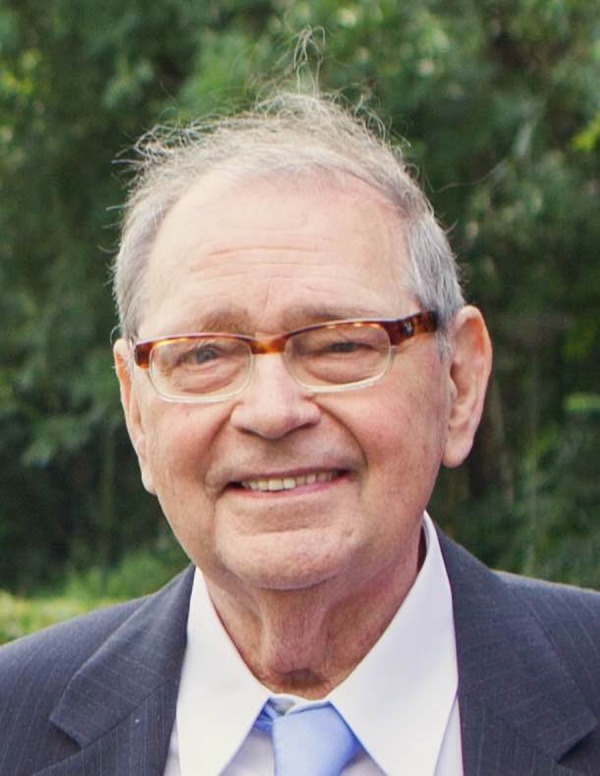
Professor Karel H. Wesseling.

In this editorial, a few researchers who knew him well briefly review some of the important contributions that Karel made to their field and how it helped in advancing science. (B.E.W.)

## Innovation at the Basis of New Medical Devices

Karel's motto in his professional life was “helping clinicians in their care for patients by providing them innovative, noninvasive concepts, methods and tools.” As physicist, engineer, and innovator, he realized that to achieve a situation where tools would actually be widely available to clinicians, major investments in research and development (R&D) and product development would be required, which would only be achieved with commercial, corporate involvement, which in turn would require intellectual property and patent protection. Karel's innovations and patents span more than four decades.

In the 1970s, the development of his extensive model of the human circulation among other things led to the first method and algorithm for the beat-to-beat computation of cardiac output from the pressure waveform, the so-called Wesseling cZ method (39), which was patented, commercially licensed, and implemented in a patient monitor.

In the 1980s, research on continuous, noninvasive finger blood pressure, building on the original volume clamp principle patent of Jan Peňáz (33), led to a series of broad physiology and technology innovations and patents (34), including the ones on the Physiocal set point (35) for the volume clamp principle, finger cuff, method of calibration, proportional pneumatic control valve, and electronic light-emitting diode driver. This unique set of intellectual property and patents again led to a commercial license and development of the Finapres 2300 patient monitor.

The major limitation of Finapres, measuring blood pressure at the finger, was solved by R&D in the 1990s, and the development of brachial blood pressure waveform reconstruction (5, 11), licensed and implemented in Finometer and with a further R&D development, was also used in the Nexfin and ClearSight patient monitor.

The R&D efforts in this period also resulted in a battery-operated, ambulatory, 24-h blood pressure device, called Portapres, which was provided to many research groups around the world but also was the basis for a series of contracts with space agencies (Deutschen Zentrums für Luft- und Raumfahrt, Centre National d'Études Spatiales, European Space Agency and National Aeronautics and Space Administration) for the development of several generations of space-qualified “specials” which were used in space shuttle missions, aboard the Russian space station Mir, and now, with Cardiopres, in routine use on the International Space Station.

Further R&D in this period led to a new concept of modeling the real-time flow waveform from pressure, resulting in the second generation method and algorithm for the beat-to-beat computation of cardiac output from the pressure waveform, the so-called Modelflow method (36), which was patented, commercially licensed and implemented in several patient monitors. More recently, the two generations formed the basis for the third generation algorithm, called CO-trek (2, 27), which is implemented in the Nexfin and ClearSight patient monitor platforms.

A series of innovations and patents have led to widely available devices and methods that have changed the world of noninvasive hemodynamic monitoring for good. When we continue the R&D and product development in this field, we stand on the shoulders of a giant. (J.J.S.)

## Waveform Filtering

The shape of the arterial pressure wave changes on its way to the periphery due to pulse wave reflections, pulse wave distortions, and resistance to pressure. During the development of Finapres, Karel realized at an early stage that peripheral and, especially, finger pressure measurements are sensitive to such effects (4, 23, 38). At the level of the finger high-frequency components, i.e., the systolic upstroke, are amplified, and low-frequency components, i.e., the diastolic pressure, are attenuated. These distortions may limit the clinical use of finger pressure measurements. They can be corrected for by using a frequency-dependent transfer function, the inverse of the formula describing the amplification toward the periphery. Waveform filtering with generalized transfer functions is most often used to reconstitute aortic pressure waves from radial artery pressure recordings (15). Karel and coworkers (5, 10) applied the technique to successfully reconstruct brachial and aortic pressure waves from finger pressure registrations, thus enabling physicians to use upper arm pressures and physiologists to study the effect of blood pressure on the heart with aortic pressures. In later years, Karel worked on further improvements of the technique by investigating ways to individualize the generalized waveform filter (41).

Karel was a physicist with a keen eye for the needs of physicians and physiologists. Across institutional borders, he collected a group of professionals consisting of physicists interested in physiology and medicine, and physicians and physiologists with a sense for physics. It has been a delight to participate under the guidance of Karel Wesseling. (W.J.W.B.)

## From Arterial Modeling to Practical Pressure Measurement

When we learned to know each other in 1969, Karel and I were both interested in the physical basis of arterial function. We discussed about the arterial windkessel model (42), where Karel was especially interested in its practical use. At that time, the quantitative measurement of arterial pressure using the finger came around: the Peňáz method (33). Karel's objective was to develop a method to continuously and noninvasively measure cardiac output from finger pressure. The first step was derivation of proximal aortic pressure wave shape and magnitude from finger pressure (23). The second step was the calculation of cardiac output from pressure, using the windkessel parameters. Under his supervision, an extensive study was carried out in our laboratory for physiology to obtain data on aortic stiffness in aging (22). These data on human postmortem aortas gave quantitative information of two important windkessel parameters, namely characteristic impedance and aortic stiffness. Knowing these parameters as a function of age allows estimation of aortic flow wave shape and cardiac output from the calculated proximal aortic pressure (27, 36). Additionally, methods were developed to calculate the sensitivity of the baroreflex acting on heart rate and on vascular resistance (3). All taken together, this resulted in practical methods, from the Finapres to the present-day Nexfin and ClearSight monitors. Karel has shown that the use of basic information on arterial function (transfer of pressure waves and arterial stiffness) can lead to the practical use of noninvasive pressure and flow monitoring. I have lost a very intelligent and wonderful personal friend. (N.W.)

## Cardiovascular Variability

Blood pressure has long been known to rise because of emotions or physical exercise and to drop during rest. With the advent of a method to measure blood pressure noninvasively on a beat-to-beat basis during daily life, this variability itself has become the subject of much research.

Earlier analysis of cardiovascular variability was restricted to that of heart rate (HRV) (16). It was demonstrated (1) that HRV virtually disappears after vagal blockade not only at the respiratory but also at the lower frequencies, thus proving that even these are not due to oscillations in sympathetic outflow. Computer modeling demonstrated that the 0.1-Hz rhythm most probably originates from blood pressure oscillations due to delays in the baroreflex-systemic resistance feedback loop (6), HRV at the same frequency being due to heart rate reflexly “riding” on blood pressure changes. The combination of such data led Malliani et al. (24) to promote the use of the LF-to-HF ratio in heart rate (LF: variance in the LF = 0.1 Hz, and HF variance at the respiratory rate) as a measure of sympathovagal balance in autonomic nervous outflow.

How can blood pressure be regulated and still exhibit the extent of variability that is observed during a normal day? Wesseling proposed the “baromodulation” hypothesis (20, 37) to explain this phenomenon. This assumes the transfer of baroreceptor input information to autonomic output itself being modulated by events outside the baroreflex. Measurement of baroreflex sensitivity on a continuous basis, a technique he developed to be used in conjunction with Finapres recordings (40), supports this hypothesis.

It was a pleasure and a privilege to have known Karel for some 35 years, in particular sharing his years at the Academic Medical Center. When he showed his experimental finger-pressure set-up with loudspeaker magnet, I never dreamed it would ever fly as high as it did, eventually. (J.M.K.)

## Autonomic Function Testing

Knowledge of the physiological mechanisms underlying disturbances in cardiovascular control mechanisms is of direct relevance for clinicians: it is the key to taking an intelligent history and understanding the appropriate emphasis for the physical examination. Only then is the doctor able to diagnose the condition and reassure a worried patient by explaining it in lay terminology. In addition, mechanistic insight is a crucial factor for the design and the evaluation of therapeutic measures.

In this context, the Finapres or volume clamp method developed by Peňáz and Wesseling (33, 35) with its ability to measure the arterial pressure in the finger noninvasively and continuously has been an enormous step forward in the evaluation of autonomic cardiovascular control (43). Monitoring of finger arterial pressure enables a clinician to study the dynamics of circulatory responses in detail. A further refinement was the calculation of beat-to-beat changes in stroke volume from the pulse wave with Modelflow (36). This allows the clinician to evaluate the hemodynamics underlying observed changes in blood pressure in terms of cardiac output and total peripheral resistance (43). This has opened new avenues of investigation in the laboratory, like the evaluation of the hemodynamic mechanisms underlying tilt table-induced syncope (32) and the effects of physical counterpressure maneuvers on a low standing blood pressure, such as leg crossing and squatting (21).

I had the good fortune to cooperate with Karel Wesseling in the Finapres evaluation studies since the early 1980s (17), the close interactions between Karel as a biomedical engineer and me as a clinician studying initial orthostatic hypotension (26), fainting in young healthy subjects (7, 8), and unusual circulatory responses in patients have been fruitful (28, 30, 31). In the process we became good friends. (W.W.)

## The Volume Clamp Method in the Epidemiologist's Tool Kit

Karel Wesseling's contributions to cardiovascular physiology are immensely important and wide ranging. He will be remembered by innumerable physiologists, anesthesiologists, autonomic nervous function specialists, and most recently, a growing number of cardiovascular epidemiologists. Karel's development of the volume clamp method for noninvasive finger arterial blood pressure measurement into a true cardiovascular “leatherman” tool has opened the field of population studies for serial hemodynamic observations. Given the gap in cardiovascular health between the wealthy and the poor, and more intriguingly between various ethnicities in the world, there is a genuine need to study the origins, for example, of the impressive differences in stroke prevalence between white and black people, or the devastating coronary atherosclerosis in people of Asian descent. Understandably, conventional risk factor analysis has been monopolizing field studies for decades. Karel has now enriched our tool kit with reproducible arterial blood pressure curves that closely follow changes in blood pressure during simple circulatory challenges, and pulse contour-derived stroke volume and vascular resistance. Systemic hemodynamics can now easily be estimated outside the laboratory and related to rates of conventional risk factors to better detect and follow these inequalities from their early start. For instance, preliminary findings in our ongoing population survey in Dutch Suriname comparing healthy Creole and Hindustani subjects show interesting differences in blood pressure regulation during changing posture: in the face of similar rises in blood pressure, in Creole subjects, resistance increases more than in Hindustani subjects, while left ventricular contractility does not change, whereas contractility drops in Hindustani subjects (F.S. Diemer, G. P. Oehlers, J. Q. Aartman, S. M. Baldew, F. A. Karamat, A. V. Jarbandhan, G. van Montfrans, L. M. Brewster, unpublished observations).

More accurate cardiovascular risk profiling, made possible by Karel's gentle, probing, independent mind, should ultimately broaden the evidence we need to obtain better-focused guidelines for those who suffer disproportionally. (G.A.v.M.)

## Noninvasive Hemodynamics in Clinical Practice

Interpretation of the heart rate and arterial pressure response to a reduced central blood volume is complex, and properly diagnosing hypovolemic shock continues to challenge the clinician since the Second World War (12, 25). Loss of 1 liter of blood or fluid does not affect arterial pressure, making it an imprecise parameter to recognize central hypovolemia (14). The blood volume is characterized not only by its size but also by its function as preload to the heart. From that perspective, a functional definition of “normovolemia” as a reference for volume treatment is by its ability to provide the heart with an appropriate central blood volume (i.e., the cardiac preload that maintains cardiac output) (13). The measurement of blood flow is so complicated that arterial pressure remains to be the monitored variable although according to Jarisch (18) more than 80 years ago *“die meisten Organe gar nicht Druck, sondern Stromvolumen brauchen* [most organs don't need pressure but flow].” By modeling aortic flow from pressure, Wesseling and coworkers (19, 36) provided clinicians with continuous information on cardiac output extracted from noninvasive arterial pressure (29). This major contribution to cardiovascular monitoring makes it possible to introduce titration of fluid administration as the cornerstone of goal-directed fluid treatment into clinical practice.

Karel was my inspiring teacher in biomedical signal analysis and my older, wise friend and companion in research in Oslo, Copenhagen, Amsterdam, and at home in The Hague with his beloved Hanny. (J.J.v.L.)

### Conclusion.

Professor Wesseling passed away on September 4, 2014, in The Hague, The Netherlands. He leaves behind his wife Hanny, their three children, and a grandson whom he unfortunately did not live to see. While we are thankful to have had the opportunity to work with Karel for so many years, we are saddened by the loss.

We will miss his original way of thinking, his wit, and his friendship. His intellectual heritage in the field of physiology and noninvasive hemodynamic monitoring will live on. (B.E.W.)

## DISCLOSURES

B. E. Westerhof and J. J. Settels are employed by Edwards Lifesciences BMEYE.

## AUTHOR CONTRIBUTIONS

B.E.W. conception and design of research; B.E.W. prepared figures; B.E.W., J.J.S., W.J.W.B., N.W., J.M.K., W.W., G.A.v.M., and J.J.V.L. drafted manuscript; B.E.W. edited and revised manuscript; B.E.W., J.J.S., W.J.W.B., N.W., J.M.K., W.W., G.A.v.M., and J.J.V.L. approved final version of manuscript.

## References

[1] Akselrod S, Gordon D, Madwed JB, Snidman NC, Shannon DC, Cohen RJ Hemodynamic regulation: investigation by spectral analysis. Am J Physiol Heart Circ Physiol 249: H867–H875, 1985. 10.1152/ajpheart.1985.249.4.H867 4051021

[2] Bogert LW, Wesseling KH, Schraa O, Van Lieshout EJ, de Mol BA, van Goudoever J, Westerhof BE, van Lieshout JJ Pulse contour cardiac output derived from non-invasive arterial pressure in cardiovascular disease. Anaesthesia 65: 1119–1125, 2010. 20860647 10.1111/j.1365-2044.2010.06511.x

[3] Borgers AJ, van den Born BJ, Alkemade A, Eeftinck Schattenkerk DW, van Lieshout JJ, Wesseling KH, Bisschop PH, Westerhof BE Determinants of vascular and cardiac baroreflex sensitivity values in a random population sample. Med Biol Eng Comput 52: 65–73, 2014. 24142561 10.1007/s11517-013-1111-0

[4] Bos WJ, Van den Meiracker AH, Wesseling KH, Schalekamp MA Effect of regional and systemic changes in vasomotor tone on finger pressure amplification. Hypertension 26: 315–320, 1995. 7635541 10.1161/01.hyp.26.2.315

[5] Bos WJ, Van Goudoever J, Van Montfrans GA, Van den Meiracker AH, Wesseling KH Reconstruction of brachial artery pressure from noninvasive finger pressure measurements. Circulation 94: 1870–1875, 1996. 8873662 10.1161/01.cir.94.8.1870

[6] De Boer RW, Karemaker JM, Strackee J Hemodynamic fluctuations and baroreflex sensitivity in humans: a beat-to-beat model. Am J Physiol Heart Circ Physiol 253: H680–H689, 1987. 10.1152/ajpheart.1987.253.3.H680 3631301

[7] de Jong-de Vos van Steenwijk CC, Wieling W, Harms MP, Wesseling KH Variability of near-fainting responses in healthy 6–16-year-old subjects. Clin Sci (Lond) 93: 205–211, 1997. 9337634 10.1042/cs0930205

[8] de Jong-de Vos van Steenwijk CC, Wieling W, Johannes JM, Harms MPM, Kuis W, Wesseling KH Incidence and hemodynamic characteristics of near-fainting in healthy 6- to 16-year old subjects. J Am Coll Cardiol 25: 1615–1621, 1995. 7759714 10.1016/0735-1097(95)00056-a

[10] Gizdulich P, Imholz BP, van den Meiracker AH, Parati G, Wesseling KH Finapres tracking of systolic pressure and baroreflex sensitivity improved by waveform filtering. J Hypertens 14: 243–250, 1996. 8728303 10.1097/00004872-199602000-00014

[11] Gizdulich P, Prentza A, Wesseling KH Models of brachial to finger pulse wave distortion and pressure decrement. Cardiovasc Res 33: 698–705, 1997. 9093542 10.1016/s0008-6363(97)00003-5

[12] Grant RT, Reeve EB Clinical observations on air-raid casualties. Br Med J 2: 293–297, 1941. 20783834 10.1136/bmj.2.4208.293 PMC2162444

[13] Harms MP, Secher NH, van Lieshout JJ Monitoring of goal-directed fluid challenge. Crit Care Med 35: 673; author reply 673–674, 2007. 17251732 10.1097/01.CCM.0000254332.29816.C6

[14] Harms MPM, van Liesthout JJ, Jenstrup M, Pott F, Secher NH Postural effects on cardiac output and mixed venous oxygen saturation in humans. Exp Physiol 88: 611–616, 2003. 12955161 10.1113/eph8802580

[15] Hirata K, Kawakami M, O'Rourke MF Pulse wave analysis and pulse wave velocity: a review of blood pressure interpretation 100 years after Korotkov. Circ J 70: 1231–1239, 2006. 16998252 10.1253/circj.70.1231

[16] Hyndman BW, Kitney RI, Sayers BM Spontaneous rhythms in physiological control systems. Nature 233: 339–341, 1971. 4940430 10.1038/233339a0

[17] Imholz BP, Van Montfrans GA, Settels JJ, Van der Hoeven GM, Karemaker JM, Wieling W Continuous non-invasive blood pressure monitoring: reliability of Finapres device during the Valsalva manoeuvre. Cardiovasc Res 22: 390–397, 1988. 3224351 10.1093/cvr/22.6.390

[18] Jarisch A Kreislauffragen. Deutsche Med Wochenschr 54: 1211–1213, 1928.

[19] Jellema WT, Wesseling KH, Groeneveld AB, Stoutenbeek CP, Thijs LG, van Lieshout JJ Continuous cardiac output in septic shock by simulating a model of the aortic input impedance: a comparison with bolus injection thermodilution. Anesthesiology 90: 1317–1328, 1999. 10319780 10.1097/00000542-199905000-00016

[20] Karemaker JM, Wesseling KH Variability in cardiovascular control: the baroreflex reconsidered. Cardiovasc Eng 8: 23–29, 2008. 18041583 10.1007/s10558-007-9046-4

[21] Krediet CT, van Dijk N, Linzer M, van Lieshout JJ, Wieling W Management of vasovagal syncope: controlling or aborting faints by leg crossing and muscle tensing. Circulation 106: 1684–1689, 2002. 12270863 10.1161/01.cir.0000030939.12646.8f

[22] Langewouters GJ, Wesseling KH, Goedhard WJ The static elastic properties of 45 human thoracic and 20 abdominal aortas in vitro and the parameters of a new model. J Biomech 17: 425–535, 1984. 6480618 10.1016/0021-9290(84)90034-4

[23] Lasance HA, Wesseling KH, Ascoop CA Peripheral pulse contour analysis in determining stroke volume. Progress Report Inst Med Phys 5: 59–62, 1976.

[24] Malliani A, Pagani M, Lombardi F, Cerutti S Cardiovascular neural regulation explored in the frequency domain. Circulation 84: 482–492, 1991. 1860193 10.1161/01.cir.84.2.482

[25] Secher NH, Van Lieshout JJ Hypovolemic shock. In: Clinical Fluid Therapy in the Perioperative Setting (2nd ed.), edited by Hahn RG Cambridge: Cambridge University Press, 2011, p. 166–176.

[26] Sprangers RLH, Wesseling KH, Imholz AL, Imholz BP, Wieling W Initial blood pressure fall on stand up and exercise explained by changes in total peripheral resistance. J Appl Physiol 70: 523–530, 1991. 2022542 10.1152/jappl.1991.70.2.523

[27] Truijen J, van Lieshout JJ, Wesselink WA, Westerhof BE Noninvasive continuous hemodynamic monitoring. J Clin Monit Comput 26: 267–278, 2012. 22695821 10.1007/s10877-012-9375-8 PMC3391359

[28] van Lieshout JJ, Imholz BP, Wesseling KH, Speelman JD, Wieling W Singing-induced hypotension: a complication of a high spinal cord lesion. Neth J Med 38: 75–79, 1991. 2030815

[29] van Lieshout JJ, Wesseling KH Continuous cardiac output by pulse contour analysis? Br J Anaesth 86: 467–469, 2001. 11573617 10.1093/bja/86.4.467

[30] van Lieshout JJ, Wieling W, Wesseling KH, Endert E, Karemaker JM Orthostatic hypotension caused by sympathectomies performed for hyperhidrosis. Neth J Med 36: 53–57, 1990. 2314521

[31] van Lieshout JJ, Wieling W, Wesseling KH, Karemaker JM Pitfalls in the assessment of cardiovascular reflexes in patients with sympathetic failure but intact vagal control. Clin Sci (Lond) 76: 523–528, 1989. 2721119 10.1042/cs0760523

[32] Verheyden B, Liu J, van DN, Westerhof BE, Reybrouck T, Aubert AE, Wieling W Steep fall in cardiac output is main determinant of hypotension during drug-free and nitroglycerine-induced orthostatic vasovagal syncope. Heart Rhythm 5: 1695–1701, 2008. 19084808 10.1016/j.hrthm.2008.09.003

[33] Wesseling KH A century of noninvasive arterial pressure measurement: from Marey to Penaz and Finapres. Homeostasis 36: 2–3, 1995.

[34] Wesseling KH, De Wit B, Settels JJ, Klawer WH On the indirect registration of finger blood pressure after Peñáz. Funkt Biol Med 1: 245–250, 1982.

[35] Wesseling KH, De Wit B, Van der Hoeven GMA, Van Goudoever J, Settels JJ Physiocal, calibrating finger vascular physiology for Finapres. Homeostasis 36: 67–82, 1995.

[36] Wesseling KH, Jansen JRC, Settels JJ, Schreuder JJ Computation of aortic flow from pressure in humans using a nonlinear, three-element model. J Appl Physiol 74: 2566–2573, 1993. 8335593 10.1152/jappl.1993.74.5.2566

[37] Wesseling KH, Settels JJ Baromodulation explains shortterm blood pressure variability. In: Psychophysiology of Cardiovascular Control. New York: Plenum, 1985, p. 69–79.

[38] Wesseling KH, Settels JJ, van der Hoeven GM, Nijboer JA, Butijn MW, Dorlas JC Effects of peripheral vasoconstriction on the measurement of blood pressure in a finger. Cardiovasc Res 19: 139–145, 1985. 3986857 10.1093/cvr/19.3.139

[39] Wesseling KH, Smith NT, Nichols WW, Weber H, De Wit B, Beneken JEW Beat-to-beat cardiac output from the arterial pressure pulse contour. In: Measurement Anaesthesia, edited by Feldman SA, Leigh JM, and Spierdijk J Leiden: Leiden University Press, 1974, p. 148–164.

[40] Westerhof BE, Gisolf J, Stok WJ, Wesseling KH, Karemaker JM Time-domain cross-correlation baroreflex sensitivity: performance on the EUROBAVAR data set. J Hypertens 22: 1371–1380, 2004. 15201554 10.1097/01.hjh.0000125439.28861.ed

[41] Westerhof BE, Guelen I, Stok WJ, Wesseling KH, Spaan JA, Westerhof N, Bos WJ, Stergiopulos N Arterial pressure transfer characteristics: effects of travel time. Am J Physiol Heart Circ Physiol 292: H800–H807, 2007. 16963619 10.1152/ajpheart.00443.2006

[42] Westerhof N, Elzinga G, Sipkema P An artificial arterial system for pumping hearts. J Appl Physiol 31: 776–781, 1971. 5117196 10.1152/jappl.1971.31.5.776

[43] Wieling W, Karemaker JM Measurement of heart rate and blood pressure to evaluate disturbances in cardiovascular control. In: Autonomic failure: A Textbook of Clinical Disorders of the Autonomic Nervous System (5TH ed.), edited by Mathias C and Bannister R Oxford: Oxford University Press, 2013, p. 290–306.

